# Forest cover associated with improved child health and nutrition: evidence from the Malawi Demographic and Health Survey and satellite data

**DOI:** 10.9745/GHSP-D-13-00055

**Published:** 2013-08-14

**Authors:** Kiersten B Johnson, Anila Jacob, Molly E Brown

**Affiliations:** a ICF International, Beltsville, MD, USA; b U.S. Agency for International Development, Forestry and Biodiversity Office, Washington, DC, USA; c NASA, Biospheric Sciences Branch, Greenbelt, MD, USA

## Abstract

Healthy forests provide human communities with a host of important ecosystem services, including the provision of food, clean water, fuel, and natural medicines. Yet globally, about 13 million hectares of forests are lost every year, with the biggest losses in Africa and South America. As biodiversity loss and ecosystem degradation due to deforestation continue at unprecedented rates, with concomitant loss of ecosystem services, impacts on human health remain poorly understood. Here, we use data from the 2010 Malawi Demographic and Health Survey, linked with satellite remote sensing data on forest cover, to explore and better understand this relationship. Our analysis finds that forest cover is associated with improved health and nutrition outcomes among children in Malawi. Children living in areas with net forest cover loss between 2000 and 2010 were 19% less likely to have a diverse diet and 29% less likely to consume vitamin A-rich foods than children living in areas with no net change in forest cover. Conversely, children living in communities with higher percentages of forest cover were more likely to consume vitamin A-rich foods and less likely to experience diarrhea. Net gain in forest cover over the 10-year period was associated with a 34% decrease in the odds of children experiencing diarrhea (*P* = .002). Given that our analysis relied on observational data and that there were potential unknown factors for which we could not account, these preliminary findings demonstrate only associations, not causal relationships, between forest cover and child health and nutrition outcomes. However, the findings raise concerns about the potential short- and long-term impacts of ongoing deforestation and ecosystem degradation on community health in Malawi, and they suggest that preventing forest loss and maintaining the ecosystem services of forests are important factors in improving human health and nutrition outcomes.

## Abstract (precis)

In Malawi, net forest cover loss over time is associated with reduced dietary diversity and consumption of vitamin A-rich foods among children. Greater forest cover is associated with reduced risk of diarrheal disease. These preliminary findings suggest that protection of natural ecosystems could play an important role in improving health outcomes.

## INTRODUCTION

Forests provide critical resources and processes that benefit human populations, known as ecosystem services, including food, clean water, fuel, natural medicines, and pollination. More than 1.5 billion people worldwide rely on forest products for their livelihoods.^1^ Yet globally, about 13 million hectares of forests are lost every year, primarily due to agricultural expansion, extraction of natural resources, and human settlement.^2^ Deforestation is a major driver of biodiversity loss, which continues unabated despite global efforts to stem this loss.^3^ According to the Board of the Millennium Ecosystem Assessment, loss of ecosystem services poses a considerable barrier to achieving the Millennium Development Goals to reduce poverty, hunger, and disease.^4^ (The United Nations Secretary-General called for the Millennium Ecosystem Assessment in 2000 to assess the consequences of ecosystem change on human well-being.)

Although all populations fundamentally depend on the natural environment, people living in rural areas of the developing world are most directly dependent on ecosystem services to meet their basic needs.^5^ As biodiversity loss and ecosystem degradation continue at unprecedented rates, there is increasing concern about potential public health impacts, especially among the world's most vulnerable populations.

More than 1.5 billion people worldwide rely on forest products for their livelihoods, but 13 million hectares of forests are lost every year.

Research on the links between human health and deforestation is limited, but a handful of studies suggest that ecosystem degradation has negative impacts on public health. For example, a Nepal study from the 1980s showed that deforestation increased women's time spent gathering essential forest products (such as fuel wood and fodder for livestock) that was significant enough to reduce the amount of time they spent on agricultural production, food preparation, and breastfeeding.^6^ Researchers in Madagascar showed, through modeling, that anemia incidence in children would increase by 29% if they no longer had access to wildlife as a food source, either due to species extinction or strict enforcement of conservation policies.^7^ Among the poorest children, anemia cases would triple. In a study of 54 health districts in the Brazilian Amazon, researchers found that a 4% increase in deforestation rates was associated with a 48% increase in malaria incidence.^8^

To better understand the potential health and nutrition impacts of deforestation, we conducted a case study of Malawi using 2010 Demographic and Health Survey (DHS) data linked, via geographic information system (GIS) points, with satellite remote sensing data on forest cover and change in forest cover over a decade's time.

## HYPOTHESIS

We hypothesized that deforestation and lower percentages of forest cover (proxies for degraded environments) result in declining ecosystem services, which then open pathways to child undernutrition and poor health.

Ecosystem services are defined as the short- and long-term benefits people obtain from ecosystems, comprising:

Ecosystems provide several benefits to people, such as food, water, flood protection, waste absorption, and crop pollination.

- **Provisioning** goods and services (the production of basic goods such as food, water, fish, fuels, timber, and fiber)
- **Regulating** services (such as flood protection, purification of air and water, waste absorption, disease control, and climate regulation)
- **Cultural** services (spiritual, aesthetic, and recreational benefits)
- **Supporting** services necessary for the production of all other ecosystem services (including soil formation, production of oxygen, crop pollination, carbon sequestration, photosynthesis, and nutrient cycling)

Conversely, we hypothesized that more biodiverse environments, represented by higher percentages of forest cover, have comparatively better capacity to provide essential ecosystem services, which then translate into improved human nutrition and health outcomes.

Our outcomes of interest include children's dietary diversity, consumption of vitamin A-rich foods, stunting, and experience of diarrheal disease. We expect these outcomes to be associated particularly with the level and quality of *provisioning* and *regulating* ecosystem services.

**Figure 1.**
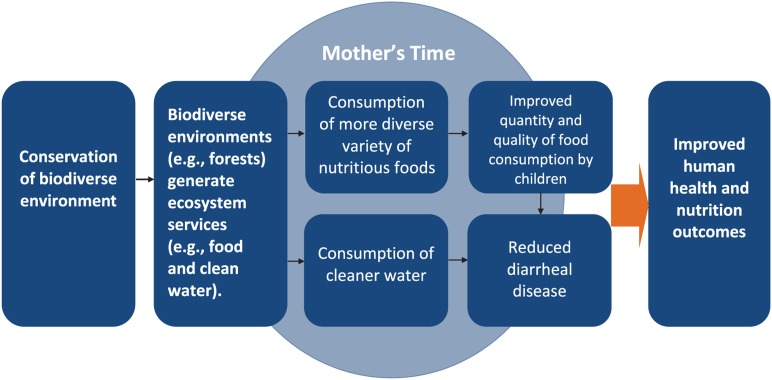
Conceptual Framework of Pathways Between Biodiverse Environments and Human Health and Nutrition Outcomes We hypothesize that biodiverse environments are better able to produce ecosystem services, such as food and clean water, which, in turn, improve the quantity and quality of food consumption by children and reduce diseases, leading ultimately to better health and nutrition outcomes. Mother's time acts as a mediating factor between biodiversity and child health and nutrition outcomes.

A comprehensive description of the complex and nonlinear relationships between the environment and human health outcomes is beyond the scope of this paper. However, our conceptual framework provides a simple illustration of the 3 hypothesized relationships that we explore in this analysis: (a) between forest cover and nutritional outcomes; (b) between forest cover and health outcomes; and (c) the mediating function of women's time on the relationship between forest cover and nutritional outcomes (Figure 1).

### Hypothesized Pathways Between Forest Cover and Nutritional Outcomes

*Provisioning* services provided by forests are an important source of food for the rural poor, who often rely on bushmeat and other non-timber forest products (NTFPs) for their protein and micronutrients. In the Congo Basin, bushmeat provides about half of people's protein intake.^9^ In a recent study of rural households in western Ghana, researchers found that residents routinely consumed a variety of forest foods, including bushmeat, plants, fruits, and snails.^10^ Residents of poorer households consumed these items more often than residents of wealthier households—on average, 5 days a week compared with 3 days a week, respectively. Furthermore, many households also sold various NTFPs at local markets, generating additional income that improved food security. In rural Malawi, researchers found that food from forests provided a safety net during times of crop failure; study participants reported that, on average, more than 50% of their meals were derived from forests during times of famine.^11^

Poor, rural residents often rely on forest foods for their nutrients.

These studies demonstrate reliance on NTFPs, particularly among the rural poor, and provide a strong basis for the hypothesis that deforestation results in decreased availability of forest foods, which in turn can lead to comparatively poorer nutritional outcomes among children as well as adults.

### Hypothesized Pathways Between Forest Cover and Health Outcomes

Populations living near intact forest ecosystems are closer to forest plants and insect life that naturally decompose human and animal waste and convert it to energy (a *regulating* ecosystem service). Forest degradation reduces these functions, which in turn degrade the ability of insect life to process animal waste.^12^

In Indonesia, a study found that residents of households living downstream from protected forested watersheds were less likely to experience diarrhea than those living downstream from unforested watersheds, demonstrating the regulating services of forests in improving quality of drinking water.^13^ Similarly, we hypothesize that children living in households close to forest cover will experience reduced odds of diarrheal disease compared with children not living close to forest cover.

Forests help to improve the quality of drinking water.

### Hypothesized Mediating Function of Women's Time on the Relationship Between Forest Cover and Nutritional Outcomes

The Nepal study^6^ demonstrated a negative association between deforestation and women's time allocation on nutrition-related functions such as food preparation and breastfeeding, subsequently limiting women's ability to provide nutrition to their children. Although we cannot test the role of women's time allocation in mediating the hypothesized relationship between forest cover and child health and nutrition outcomes in this study, we believe it is important to represent this mediating function in the conceptual model, given the well-demonstrated centrality of women's role in ensuring child health and nutrition outcomes.

## DATA AND METHODS

### Study Site Selection

We selected Malawi as our study country given its very high rates of deforestation, child undernutrition, and child mortality, as well as the direct dependence of the majority of the population on ecosystem services. Malawi has lost almost 600,000 hectares of primary forest between 1990 and 2005, with regional deforestation rates as high as 3.4% per year.^14^ Over 80% of Malawi's population is rural and highly dependent on natural resources for food, fuel, and maintenance of livelihoods.^14^-^15^ Forty-seven percent of children are stunted, and the under-5 mortality rate is 112 deaths per 1,000 live births.^16^

### Data

#### Demographic and Health Surveys Data

We obtained nutritional status and dietary consumption data, as well as other individual-level characteristics, from the 2010 Malawi DHS.

The DHS are a key source of comparative quantitative data across developing countries on demographic and health indicators, including for reproductive health, maternal and child health, and nutritional status of women and children. They are nationally and sub-nationally representative household surveys with large sample sizes and detailed data obtained by interviewing women of reproductive age (15–49 years old) to obtain information about their children and other household members. The data also include information on household and other socioeconomic characteristics of sampled women and their households.

The DHS are implemented using a stratified 2-stage cluster sampling design. In Malawi, more than 23,000 households participated in the 2010 survey (household response rate: 98%; individual women's response rate: 97%).^16^ For more information about the DHS, see www.measuredhs.com.

#### Satellite Remote Sensing Data

We used 2 types of satellite remote sensing variables in this analysis: the Vegetation Continuous Fields (VCF) product^17^ and the Normalized Difference Vegetation Index (NDVI).^18^ A full technical description of the satellite remote sensing data sets used in this analysis is available in the supplementary Appendix; the variables are described below.

#### GPS Data and Displacement

During DHS fieldwork activities, surveyors collected the GPS coordinates for the center of the populated area surveyed (cluster centroid) using handheld GPS units. Of importance to this analysis is that the GPS coordinates are displaced to ensure respondent confidentiality.^16^ The displacement is randomly applied so that rural points contain a minimum of 0 km and a maximum of 5 km of positional error. Urban points contain a minimum of 0 km and a maximum of 2 km of error. A further 1% of the rural sample points are offset a minimum of 0 km and a maximum of 10 km.

We overlaid DHS data with average NDVI data derived from NASA's Moderate Resolution Imaging Spectroradiometer (MODIS) (a key satellite instrument that views the Earth's surface) at each DHS cluster centroid for 2010. We used a similar procedure with the VCF product to link the percent forest cover variable and the change in forest cover variable to the 2010 Malawi DHS data set.

We chose to aggregate the satellite data to 5 km resolution to ensure that even after displacement of DHS GPS coordinates, environmental variability and tree cover were accurately represented for each DHS cluster. Displacement errors were below the spatial resolution of the satellite remote sensing.

### Variable Definitions

#### Dependent Variables

Our analysis explored 4 dependent, dichotomous variables: severe stunting, dietary diversity, consumption of vitamin A-rich foods, and diarrhea.

1. **Severe stunting:** The height, weight, and age of all children under age 5 were collected during DHS fieldwork using UNICEF SECA weight scales and Shorr height boards; these data were then used to calculate measures of child stunting. Stunting, or height-for-age, reflects the long-term nutritional status of a child, indicating impacts of both nutritional deficiencies and bouts with illnesses. Children with height-for-age z-scores that are less than -3 standard deviations from the median of the reference population are considered to be severely stunted. We coded severely stunted children with a “1” and all remaining children with a “0.”
2. **Dietary diversity:** The DHS instrument collects 24-hour recall data on dietary consumption for the most recently born child under the age of 3. Our definition of dietary diversity was based on the Minimum Dietary Diversity indicator^19^ defined by an expert working group led by the World Health Organization–that is, the proportion of children 6–23 months of age who receive foods from 4 or more of the following food groups: (a) grains, roots, and tubers; (b) legumes and nuts; (c) dairy products (milk, yogurt, and cheese); (d) flesh foods (meat, fish, poultry, and liver/organ meats); (e) eggs; (f) vitamin-A rich fruits and vegetables; and (g) other fruits and vegetables. For our definition, we also included breast milk as one of the food groups, given its importance as a source of nutrition for children and that breastfeeding practices are likely to be impacted by deforestation. We also included all children under age 3 in our analysis, given that many children are given foods other than breast milk prior to 6 months of age, despite the recommendation that infants be exclusively breastfed until that age. We coded children who consumed foods from 4 or more food groups with a “1” and all remaining children with a “0.”
3. **Consumption of vitamin-A rich foods:** Data on consumption of vitamin-A rich foods were also drawn from the 24-hour recall data on dietary consumption for the most recently born child under the age of 3, described above. All children under age 3 were included in the analysis, and we coded children who consumed vitamin-A rich foods (fruits or vegetables) with a “1” and all remaining children with a “0.”
4. **Diarrhea:** In DHS surveys, mothers of children under age 5 are asked whether their children have experienced a bout of diarrhea in the past 2 weeks. We coded children whose mothers reported in the affirmative with a “1” and all remaining children with a “0.”

#### Independent Variables of Interest

We calculated our key independent variables from the remotely sensed VCF data product mentioned earlier. The VCF contains proportional estimates for vegetative cover types; it shows how much of a land cover, such as “forest” or “grassland,” exists on the land surface.

The VCF product was averaged to 5 km and was used to generate 2 independent variables:

1. Percentage of forest cover associated with each DHS sampling cluster (0–9%, 10–19%, 20–29%, 30–39%, 40–49%, and 50–59% forest cover)
2. Another categorical variable reflecting changes in forest cover over a decade (no change, net forest loss, and net forest gain) for the sampled DHS cluster

The variable reflecting decadal change in forest cover leverages the longitudinal nature of the satellite remote sensing data. Adding this temporal dimension to the analysis strengthened our study considerably over using only a static measure of forest cover taken at the time of the survey, allowing us to test, in effect, the relationship between forest change over time (loss or gain) and our outcomes of interest.

#### Control Variables

Variables controlled for in the analysis include child's age (grouped into 6-month categories), mother's education (none, primary, secondary or higher), urban or rural residence, household wealth quintiles, number of years resident in the current location, and a remotely sensed vegetation index (NDVI). The model for diarrhea includes whether the household has access to a safer toilet facility and water source. Selection of variables was driven in part by data availability, given that we are analyzing secondary source data.

The variable on *mother's education* is a self-reported measure reflecting the mother's highest education level achieved at the time of the survey. Respondents are asked to report the level of highest achievement and the grade achieved within that level. Our analysis used a recoded categorical variable that reflects attainment of: no education, primary education, and secondary education or higher. Mother's education is related to a child's height-for-age^20^: mothers who are better-educated may be more knowledgeable about nutrition and optimal child care behaviors, enabling them to improve health outcomes for their children.

Relative *household wealth* is measured using an asset-based index: data on household asset ownership collected during the DHS household interview are dichotomized (yes/no to indicate ownership of each asset) and entered into a statistical procedure known as principal components analysis (PCA) that assigns a weight to each asset. The asset values for each household are then summed taking into consideration the weights, and a total household score is given. The household population is then divided into quintiles on the basis of the score given to their household.^21^

The *NDVI* is a satellite remote sensing variable that quantifies the concentrations of green leaf vegetation on the ground, providing an integrated estimate of vegetation health and a means of monitoring changes in vegetation over time. We used NDVI as a proxy control for the growing conditions of the area during the growing season immediately prior to administration of the DHS survey; it is the average NDVI for December, January, and February prior to DHS implementation (June through November 2010). When vegetation is brown because it is the dry season, or because the climate is unusually dry, the NDVI is low. When the climate is wet, and the grass, trees, and other land cover is green, the NDVI is high. There is 1 NDVI measurement associated with each DHS sampling cluster.

### Statistical Methods

We conducted multivariate analyses using unweighted binomial logistic regressions to examine the correlation between our independent variables of interest (forest cover or decadal change in forest cover) and our selected dichotomous outcome variables (severe stunting, dietary diversity, consumption of vitamin-A rich foods, and experience of diarrhea), while controlling for confounding factors for which data were available. Logistic regression does not impose restrictive normality assumptions on predictors. The results are expressed in odds ratios that indicate the associations between the independent and dependent variables without attributing causality.

Only the most recent birth for each mother interviewed was included in the analysis to avoid intra-household correlations in the selected outcomes. The unweighted number of cases contributing to each analysis was as follows: dietary diversity (n = 9,166), consumption of vitamin A-rich foods (n = 9,166), experience of diarrhea (n = 12,831), and stunting (n = 3,173).

## RESULTS

**Figure 2.**
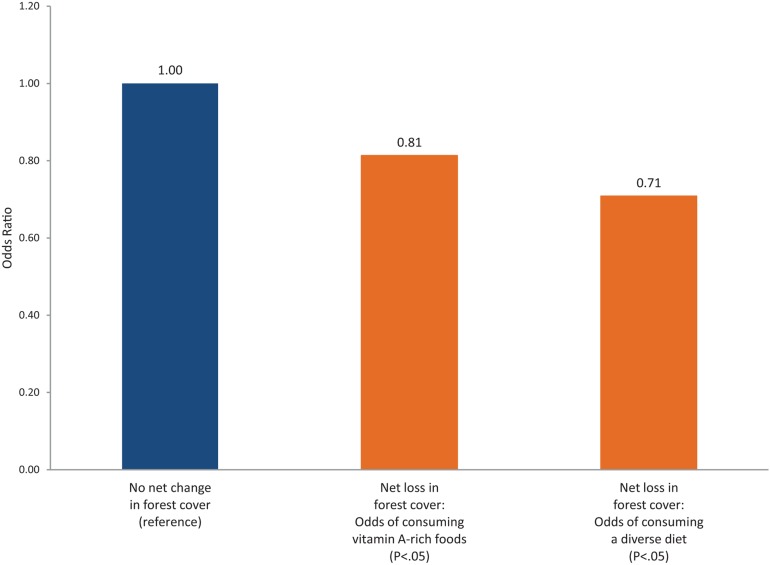
Logistic Regression Results: Net Loss of Forest Cover Reduces the Odds That a Child Will Consume Vitamin A-Rich Foods and Have a Diverse Diet

We found that children living in DHS clusters with a net loss of forest cover over the past decade were 19% less likely to have a diverse diet and 29% less likely to consume vitamin A-rich foods than children living in clusters with no net change in forest cover (Figure 2). These differences were statistically significant.

Children living in areas with a net loss of forest cover were significantly less likely to have a diverse diet and to consume vitamin-A rich foods than children living in areas with no net change in forest cover.

**Figure 3.**
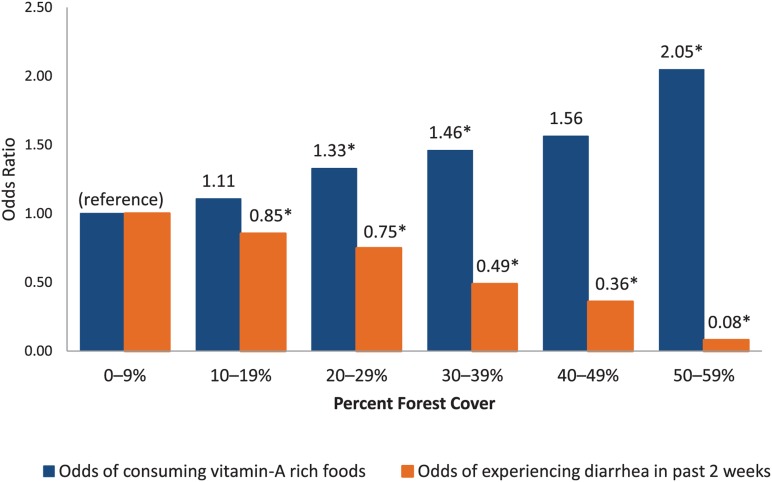
Logistic Regression Results: Greater Forest Cover Increases the Odds That a Child Will Consume Vitamin A-Rich Foods and Decreases the Odds That the Child Will Experience Diarrhea * *P*<.05 (statistically significant).

Conversely, children living in communities with higher percentages of forest cover were more likely to consume vitamin A-rich foods and less likely to experience diarrhea (Figure 3). Children living in clusters with a net gain in forest cover from 2000 to 2010 were 34% less likely to experience diarrhea (*P* = .002). No statistically significant associations were found between the percentage of forest cover variable and child stunting, but there was a marginally statistically significant association between net gain of forest cover and stunting (*P* = .058). Odds ratios are presented in the Table.[Fig f04]

**Figure f04:**
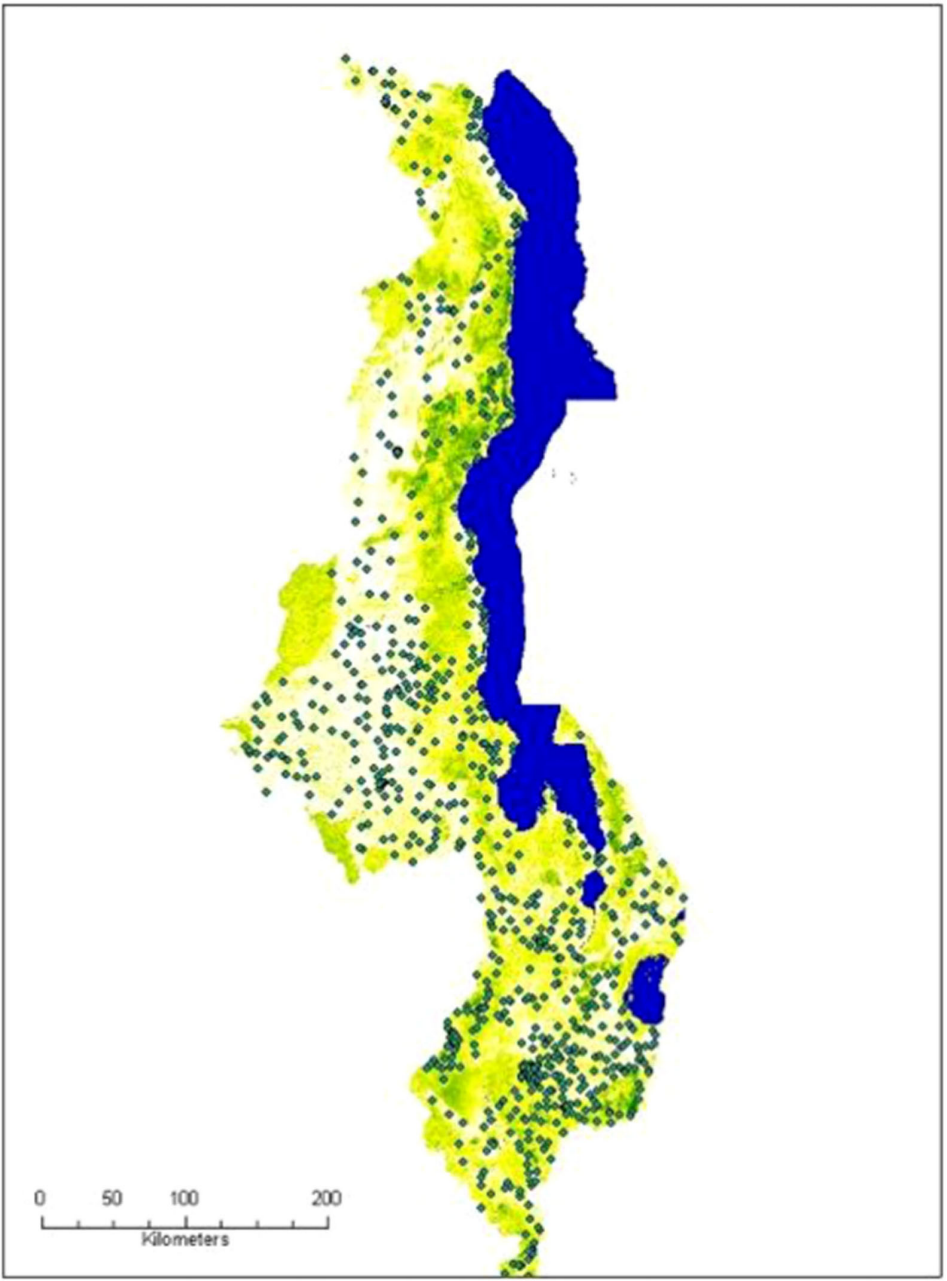
Map of Malawi showing 2010 forest cover overlaid with sampling clusters from the 2010 Malawi DHS.

**TABLE.** Logistic Regression Results: Adjusted Odds Ratios for Child Health and Nutrition Outcomes Associated With Forest Cover, Malawi 2010

Forest cover-related independent variables	Severe Stunting^a^				Dietary Diversity^b^				Vitamin A-Rich Foods^b^				Diarrhea^b^			
	Model 1		Model 2		Model 1		Model 2		Model 1		Model 2		Model 1		Model 2	
	*P* Value	OR	*P* Value	OR	*P* Value	OR	*P* Value	OR	*P* Value	OR	*P* Value	OR	*P* Value	OR	*P* Value	OR
**Deforestation, 2000–2010 (ref: no change)**	.16				.14		–	–	.002		–	–	.003		–	–
Net loss of forest cover	.64	1.113	–	–	.049	.813	–	–	.003	.708	–	–	.06	.822	–	–
Net gain of forest cover	.058	1.647	–	–	.56	.930	–	–	.16	1.201	–	–	.002	.658	–	–
**Forest cover (ref: 0–9%)**			.47		–	–	.13		–	–	.01		–	–	<.001	
10–19%	–	–	.58	1.080	–	–	.52	.961	–	–	.13	1.106	–	–	.008	.854
20–29%	–	–	.25	1.283	–	–	.08	1.188	–	–	.006	1.328	–	–	.003	.748
30–39%	–	–	.14	1.665	–	–	.34	1.177	–	–	.03	1.459	–	–	<.001	.487
40–49%	–	–	.58	.700	–	–	.75	.908	–	–	.15	1.562	–	–	.008	.358
50–59%	–	–	–	–	–	–	.08	1.736	–	–	.02	2.046	–	–	<.001	.079

Abbreviations: OR, Odds Ratio.

*P* values ≤ .05 were considered statistically significant.

a Controls included child's age, previous birth interval, mother's education, urban residence, household wealth, migration status, and Normalized Difference Vegetation Index (NDVI).

b Controls included child's age, mother's education, urban residence, household wealth, migration status, and NDVI.

The health and nutrition outcomes we found to be associated with variation in forest cover have serious implications for children's growth and development. Dietary diversity is often used as an indicator of adequate dietary intake of micronutrients.^22^ Inadequate intake of vitamin A can lead to deficiency, which is estimated to impact 60% of young children in Malawi.^23^ Vitamin A deficiency is associated with poor vision and increased susceptibility to infectious diseases such as malaria and diarrhea, with consequent mortality.^23^-^24^ Diarrhea in children in developing countries is a leading cause of mortality; a recent study estimated that up to 25% of deaths in young children in Africa can be attributable to the disease.^25^

## DISCUSSION

Our results demonstrate an association between forest cover and selected nutrition-related outcomes. A study in 3 villages in southern Malawi found that, on average, almost two-thirds of households sampled consumed wild fruit, vegetables, and mushrooms from the forest, and almost one-quarter consumed bushmeat to supplement their diets.^26^ Nearly 70% of study participants were dependent on wood collected from forests for cooking.

The dependence of these rural populations on forest products is consistent with our finding that dietary diversity and consumption of vitamin A-rich foods decrease with net decadal loss in forest cover. With respect to increased forest cover over time, we did not find a positive association with dietary diversity, and actually found a marginally significant positive relationship with severe stunting. It is our understanding, based on expert observations from Malawi, that reforested areas tend to be plantations with monocultures of non-native trees. Such areas may not provide the same diversity of animal and vegetable nutrition as native forests. Additionally, access of local populations to reforested areas tends to be more restricted than to native forests. Such reforested areas usually are privately held (for example, in the case of tea plantations) or strictly controlled by a village committee or individual. In contrast, while native forests are tightly regulated by law, in practice they are heavily accessed and used. Reduction in access to NTFPs from such reforested areas could also lead to poorer nutritional outcomes for nearby communities. Further research is needed to support these hypotheses, however.

Forest cover is significantly associated with reduced child diarrheal disease.

Our results also demonstrate an association between forest cover and reduced child diarrheal disease, supporting the hypothesis that forests play an important provisioning role, specifically in terms of water availability and/or quality, 2 factors that impact diarrheal risk in developing countries.^27^ Forests help to maintain healthy watersheds, prevent soil erosion, and filter impurities from water, all of which positively impact water availability and quality.^28^ Our finding that an increase in decadal forest cover reduces diarrhea risk suggests that tree plantations, which are the primary source of increasing forest cover in Malawi, may be as effective as older forests with regard to this particular health outcome.

In 2008, the Maternal and Child Undernutrition Study Group estimated that co-exposure of nutrition-related factors, such as vitamin A and iron deficiency, were together responsible for about 35% of child deaths worldwide and for 11% of the total global disease burden.^29^ The authors noted that the evidence makes a compelling case for the urgent implementation of interventions to reduce the various forms of undernutrition or to ameliorate their consequences.

Protecting natural ecosystems could be a sustainable and effective way to improve child nutrition and health.

Our results provide preliminary evidence suggesting that an equitable, sustainable, and effective way to improve child nutrition—and hence, survival—outcomes could include ensuring the integrity of natural ecosystems. Such an integrated approach would result in ecosystems that are healthy enough to deliver the services that local communities depend on, including foods with high nutrient bioavailability and clean water, which is essential in preventing diarrheal disease.

Given the limitations of the currently available data, it was possible to demonstrate only associations, not causal relationships, in this analysis. To establish the causal pathways and mechanisms responsible for the demonstrated associations between forest cover and child health and nutrition outcomes in Malawi, further research is needed, involving collection of accurately geo-referenced, longitudinal population and health data and women's time allocation data, as well as concomitant collection and testing of water samples for quality and use of newly-available 30-meter remote sensing data.
